# The evolution of signaling and monitoring in plant−fungal networks

**DOI:** 10.1073/pnas.2420701122

**Published:** 2025-01-21

**Authors:** Thomas W. Scott, E. Toby Kiers, Stuart A. West

**Affiliations:** a Department of Biology, University of Oxford, Oxford OX1 3SZ, United Kingdom; b School of Biology, University of St Andrews, Dyers Brae, St Andrews KY16 9ST, United Kingdom; c Amsterdam Institute for Life and Environment, Vrije Universiteit Amsterdam, Amsterdam 1081 HV, the Netherlands; d Society for the Protection of Underground Networks, SPUN, Dover, DE 19901

**Keywords:** evolutionary theory, signaling, social evolution, plant-fungal networks, cooperation

## Abstract

Experiments have shown that when one plant is attacked by a pathogen or herbivore, this can lead to other plants connected to the same mycorrhizal network up-regulating their defense mechanisms. It has been hypothesized that this represents signaling, with attacked plants producing a signal to warn other plants of impending harm. We examined the evolutionary plausibility of this and other hypotheses theoretically. We found that the evolution of plant signaling about an attack requires restrictive conditions, and so will rarely be evolutionarily stable. The problem is that signaling about an attack provides a benefit to competing neighbors, even if they are kin, and so reduces the relative fitness of signaling plants. Indeed, selection is often more likely to push plant behavior in the opposite direction—with plants signaling dishonestly about an attack that has not occurred, or suppressing a cue that they have been attacked. Instead, we show that there are two viable alternatives that could explain the empirical data: 1) the process of being attacked leads to a cue (information about the attack) which is too costly for the attacked plant to fully suppress; 2) mycorrhizal fungi monitor their host plants, detect when they are attacked, and then the fungi signal this information to warn other plants in their network. Our results suggest the empirical work that would be required to distinguish between these possibilities.

Mycorrhizal fungi form symbiotic associations with plant roots, trading nutrients such as phosphorous and nitrogen for plant-derived carbon. These fungi form physical networks of mycelium that can connect roots of different plants and act as potential routes for signaling between those plants (1–7). Several laboratory experiments have provided clear evidence that, when one plant in a mycorrhizal network is attacked, this leads to other plants in the network up-regulating their defense mechanisms. For example, when a tomato plant is infested with a leaf chewing caterpillar, tomato plants connected to the same network will increase their production of defense enzymes (8). It has been hypothesized that this pattern represents a “warning signal” in which the attacked plant actively signals to other plants using chemicals transported via the mycorrhizal network. This work has even fueled narratives in the media that forest trees use mycorrhizal networks to warn other trees of impending danger (9, 10).

However, the evolutionary plausibility of this signaling hypothesis remains unclear (2, 11–24). A signal is defined as “any act or structure that alters the behavior of other organisms, which evolved owing to that effect, and which is effective because the receiver’s response has also evolved” (25, 26). Consequently, signaling is a form of cooperation, that is only favored when it provides a benefit to both the sender and the receiver (25). Otherwise either the sender would not be selected to signal, or the receiver would be selected to ignore the signal. Plants compete with neighbors for resources such as sunlight and nutrients, and so helping a neighbor could be costly to a potential signaler (27). Neighboring plants could be relatives, which could provide a kin-selected benefit of signaling, but competition between relatives can reduce or even negate any benefit of helping relatives (28–31). For instance, a low migration rate could cause relatives to signal to each other, leading to a kin-selected benefit, but it could also cause relatives to compete with each other, negating this benefit. Consequently, it is not clear whether signaling about attack would be evolutionarily stable between neighboring plants who are both relatives and competitors.

In addition, there are at least two alternatives to plant signaling that could possibly explain the experimental data (Fig. 1) (32). One possibility is that the neighboring plants could be detecting a cue that another plant is being attacked (27). A cue is defined by when a receiver uses some feature of the sender to guide their own behavior, but this feature has not evolved for that purpose (26). An example of a cue is when a mosquito searching for a mammal to bite will fly up wind if it detects carbon dioxide (26). Mosquitos use carbon as a cue of the presence of a source of blood, but mammals do not produce carbon dioxide to signal their presence to mosquitoes (getting bitten is costly!). All that is required for a cue of plant attack is that the damage caused by attack causes the attacked plant to produce something, such as a released chemical (volatile) (33). The production of cues of herbivore attack may be unavoidable (34).

A second possible alternative explanation of the experimental data is that the fungus can detect when a plant is being attacked and that it then signals this information to the other plants to which it is connected (13). This would therefore involve fungi “monitoring” plants to become aware of cues of herbivore attack, and it would be the fungus—not the attacked plant—that is signaling. Fungi could be selected to monitor and signal to plants in their network because they actively trade resources with those individuals, and so could benefit from helping keep them in better condition, to make better trading partners (27, 35, 36).

The difference among these hypotheses matters because they represent different evolutionary outcomes, imply that information transfer is favored for different reasons, and require different empirical tests (26, 37, 38). With plant signaling, the problem is to determine how both the sender and receiver benefit. What conditions would be required for honest signaling to be evolutionary stable, and how could this seemingly altruistic act of warning neighbors be explained in the context of plant–plant competition? Could plants even be favored to signal dishonestly to harm competitors? In contrast, with a cue, we would need to ask what stimulates the cue to be produced? If the cue provides a benefit to neighbors, then does this cue impose a cost to the producer, by aiding their competitors? Why don’t plants suppress a cue? Finally, in the case of mycorrhizal monitoring, we would need to ask why would the fungus be selected to monitor and then produce a signal in response? And what exactly is being monitored?

We investigated these different hypotheses theoretically by applying kin selection and signaling theory to the question of herbivory information transfer between plants. Our aim was to determine their evolutionary plausibility, as well as define the empirical work required to test among them. We first consider selection on plants, examining whether: a) an attacked plant would be selected to signal that it had been attacked; b) plants can be selected to signal dishonestly about attack to harm competitors; and c) plants can be selected to suppress a cue that they are being attacked. We then examine selection on mycorrhizal fungi and ask whether they can be selected to monitor plants, to determine whether they are being attacked, and then signal that information to neighboring plants.

## Results

We constructed a series of theoretical models to examine selection on signals and cues from the perspective of both plants and mycorrhizal fungi. We constructed deliberately simple models which are easy to interpret and can be applied across diverse species (39–41).

### Plant Signaling Model

We first examined whether an attacked plant can be selected to produce a warning signal. We assume an infinite population of individual plants, split into patches (demes) of size *N* (infinite island model) (42). Each generation, with probability *P*, the population is attacked, for instance by a herbivore or pathogen (with probability 1-*P*, the population is not attacked). In generations where the population is attacked, a random individual on each patch, *i*, is initially attacked and suffers a fecundity cost of *d*. This individual then invests *x_i_* into the production of a signal, resulting in an additional fecundity cost of *cx_i_*, where *c* is the marginal cost of signal production.

The signal produced by the initially attacked individual is transferred to the other *N*-1 individuals on the patch (receivers). We make no assumptions about how the signal is transferred, so it may be transferred through a common mycorrhizal network, the air, or any other mechanism (17, 34). The signal warns the receivers that an attack is imminent. Consequently, the receivers can prepare for being attacked, and defend themselves, meaning they suffer a reduced fecundity cost of being attacked. We assume that the timing of herbivore attack in a given generation is not predictable, which means that individuals cannot prepare themselves for herbivore attack unless they have received a warning signal (43, 44). The extent to which signal receivers, *j*, respond to the signal, preparing for herbivore attack, rather than ignoring the signal, is given by *y_j_*. Preparation for being attacked (defense) incurs a fecundity cost of *sx_i_y_j_*, where *s* is the marginal cost of defense, but it reduces the cost of being attacked, which is now given by *d(1-x_i_y_j_*). Note that *x_i_* (signal investment by the signaler) features in these costs to the signal receiver because, to respond to a signal (mediated by *y_j_*), there needs to be a signal there to respond to (mediated by *x_i_*), hence why *x_i_* and *y_j_* are multiplied together. We assume that the cost of defense is less than being attacked (*s < d*), which ensures that individuals are favored to defend themselves against attack (rather than let themselves be damaged).

We then allow individuals to produce offspring (juvenile haploid clones) in proportion to their fecundity. A random sample of *N* juvenile individuals are chosen, for each patch, to survive and form the next adult population (local population regulation). This generates competition between juveniles to obtain a spot on the patch to grow into an adult (SI Appendix, Appendix O). After population regulation, a proportion of the new adult population, *m*, migrate to different and random patches. The remaining proportion, *1-m*, remain on their local patch. This lifecycle then iterates over many generations until an evolutionary end point is reached. In SI Appendix, Appendix L, we determined the equilibrium (ESS) levels of signaling investment (x*) and signal response (y*).

#### Signaling is not evolutionarily stable

**Fig. 2:**
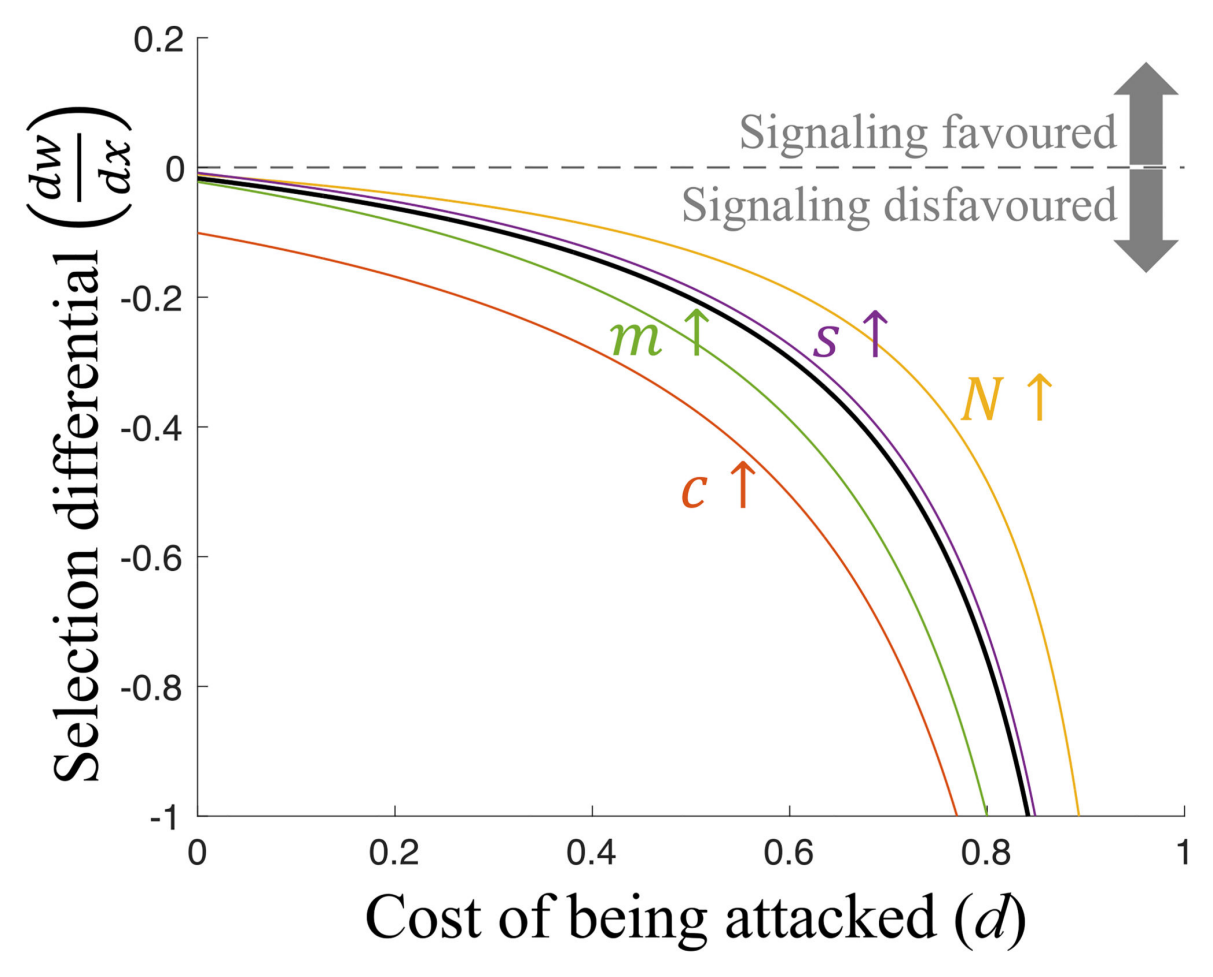
Plant signaling is not evolutionarily stable. Signaling is favored if plant fitness (*w*) increases with signaling investment (*x*), in other words, if the selection differential $\frac{dw}{dx}$ is positive. The solid black line is a reference line that plots the selection differential for a given set of parameter values. The black line never goes above zero, showing that, for our set of parameter values, the selection differential is negative, meaning signaling is disfavored. Each colored line plots the selection differential when one parameter (annotated) is changed to a higher value. The colored lines do not lie on top of the black line, but like the black line, they never go above zero. This illustrates that changing parameter values can quantitatively adjust the intensity of selection on signaling, but it cannot make the selection differential positive. Signaling is therefore disfavored for all parameter combinations. We assumed: (parameter values used for the black line) *N* = 3 (group size), *c* = 0.1 (cost of signaling), *s* = 0 (defense cost), *m* = 0.3 (migration rate); (alternative parameter values used for the colored lines) *N* = 7, *g* = 1, c = 0.6, *s* = 0.05, *m* = 1; *x* = 0, *y* = 1, *z* = 0 (signals initially rare, honest and responded-to).

We found that individual plants were not favored to produce warning signals (x*=0) (Fig. 2). Any tendency to honestly signal an impending attack will ultimately be removed by natural selection, to avoid providing a benefit to competitors. Honest signaling is a helping behavior, that increases the fitness of social partners by warning them about an impending attack. Helping can be potentially favored if it is directed toward relatives that share genes for helping, termed kin selection (28, 45). When the migration rate (*m*) is lower, individuals on a patch will be more closely related. However, a lower migration rate also means that the individuals receiving help are in stronger local competition with the helper, which negates the benefit of helping relatives (29). Put simply, there is no benefit in helping one’s relative if this comes at an equal cost to another relative (30, 46, 47). We showed in Appendices L, M, and O that this result also holds in the following scenarios: population regulation occurs after migration, rather than before; plants signal even if they are not the first plant on the patch to be attacked (obligate versus facultative signaling); competition affects fecundity rather than survival.

#### Alternative population structures

Our model assumed a simple life history where offspring (seeds) can disperse after reproduction. This is reasonable for plants, where the lack of movement after they have started growing means that any neighbors potentially helped will tend also to be competitors (48–53). We also assumed that the parameters of the life cycle such as the migration rate determined both the relatedness (genetic similarity) between different individuals on a patch and who competition occurs between. As an alternative approach, we can construct an “open” model that detaches these model parameters and keeps them as free variables (54). While this can be artificial, it can suggest the kind of conditions that would be required for honest signaling to be favored. We showed in SI Appendix, Appendix M that honest signaling can be favored when signalers are highly related to signal receivers but not to their competitors. Although theoretically this is possible, there may not be many scenarios in which low dispersal could lead to high relatedness among interacting plants, but not local competition (48, 49, 51–53). An alternative possibility is that there is a way to preferentially signal to related plants (kin discrimination) (55, 56). We show in SI Appendix, Appendix E that kin discrimination can allow honest warning signals to be evolutionarily stable. However, the extent to which plants can discriminate kin, especially with warning signals, is a matter of empirical debate (32, 57, 58). Recent theoretical work has shown how kin discrimination could be favored if herbivory information is transferred through volatiles (50). Other hypotheses for honest signaling that could be tested empirically include certain forms of generation overlap; or a private (direct) benefit of helping to reduce the local population of herbivores (28, 54, 59).

#### Can dishonest signaling be favored?

Our model also allowed us to investigate the opposite of honest signaling—whether plants could be favored to produce dishonest signals, where they signaled an attack when this had not happened. This could potentially benefit the dishonestly signaling plant if it sufficiently reduced the fecundity of their local competitors. In generations where the population is not attacked, a random individual on each deme, *i*, is given the opportunity to signal an attack even though no attack had occurred (dishonest signaling). This individual invests *cx_i_z_i_* into signal production, where *z_i_* denotes dishonesty. The receivers, *j*, consequently suffer a defense cost of *sx_i_y_j_z_i_*. In SI Appendix, Appendix L, we determined the ESS level of signal dishonesty (z*) and how it influences signal response (y*).

We found that individuals could be favored to produce dishonest signals, in which they signaled an attack even when none had occurred. Plants could gain a benefit from dishonest signaling because it harms their local competitors. However, this leads to selection on the receiver plants to ignore signals, and so dishonest signaling would only be transient, not evolutionarily stable. Dishonest signaling has not been empirically observed, but it has also not been tested for. More generally, dishonest signals can in principle be stable within signaling systems when they are at a low frequency, and so do not completely remove the benefit of responding to signals. For example, fork-tailed drongos produce alarm calls to signal to meerkats when a predator is approaching; but also occasionally produce false alarm calls, in the absence of a predator, to steal food left by the fleeing meerkats (60, 61).

### Cue-Suppression Model

We then examined the case where a plant produces a cue when it is being attacked. This would include chemicals that are produced or released by damage from herbivores or pathogens, and which are transmitted by any route, including the air or fungal network (62–75). The production of a cue could provide another explanation for the experimental data showing upregulation of defenses by the neighbors of attacked plants. Can the plant be selected to suppress this cue? (33) We test this by modifying the assumptions of the previous model so that now, after an individual is attacked, it releases a cue rather than a signal. The cue indicates to the other *N*-1 individuals on the patch (receivers) that an attack is imminent. However, we assume that the attacked plant may pay a cost to suppress the cue, to stop the receivers from learning that an attack is imminent. So now, *x_i_* denotes investment into cue suppression, rather than signaling. We assume that plants respond to cues by up-regulating their defenses. In SI Appendix, Appendix G, we determined the ESS level of cue suppression (x*).

#### Cue suppression is evolutionarily stable

**Fig. 3:**
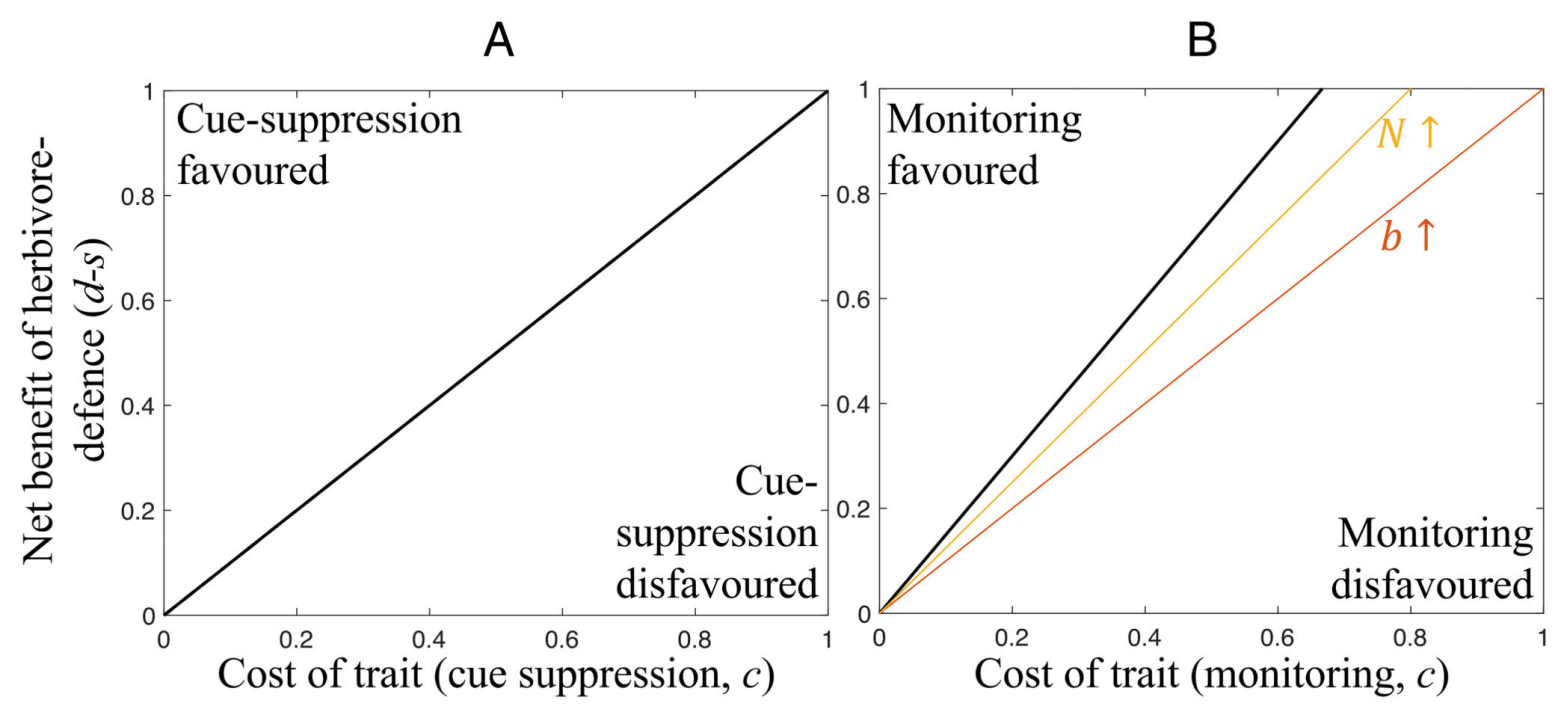
The evolution of cue suppression and monitoring. Traits are favored above the lines and disfavored below them (i.e., the lines mark the boundary where the trait changes from being favored / disfavored, for a given set of parameter values). Solid black lines show boundaries that arise with a given set of parameter values. Colored lines show boundaries that arise if one parameter is changed to a higher value. A flatter (lower gradient) boundary line implies that the trait is favored over a larger range of parameter values. Therefore, if a colored line is flatter than the black line, this implies that an increase in the value of the annotated parameter causes the trait to be favored more permissively (i.e., over a greater range of parameter combinations). (*A*) Suppression of cues of herbivore presence is favored when the net benefit of herbivore defense (*d*-*s*) is greater than the trait cost (*c*). (*B*) Mycorrhizal monitoring and signaling is more likely to be favored when the benefit of high-quality trade partners (*b*), *N* (group size), and *d* (attack cost) are high; *s* (defense cost) and *c* are low. We assumed (*A*) any value for *N*; (*B*, reference values) *N* = 3, *b* = 1; (*B*, alternative values) *N* = 5, *b* = 1.5.

We found that individuals could be favored to suppress cues (information) of an attack (Fig. 3A). Plants can gain a benefit from cue suppression because it harms their local competitors (by avoiding helping them). Cue suppression is favored when the benefit incurred due to competitors being less able to defend themselves against the attack is smaller than the cost of suppressing the cue (Fig. 3A). More generally, we showed in SI Appendix, Appendix H that cue suppression is favored with low relatedness between individuals on a patch and local competition. However, since there are many situations in which complete cue suppression is not favored, the detection of a cue that another plant is being attacked remains an evolutionarily viable explanation for the experimental data (27). For instance, in nature, it may be too costly or even impossible to suppress all cues of herbivore attack, given the sheer volume and diversity of such cues, passed through the air or fungal networks (34, 76).

### Fungal Monitoring Model

Finally, we examine selection on mycorrhizal fungi and ask whether they can be selected to monitor plants, to determine whether they are being attacked, and then signal that information to the other plants they are connected to (13). To examine this, we modify the assumptions of the first “plant signaling” model, so that now, as well as the (infinite) population of plants, we additionally model an infinite population of fungi.

The population is split into patches comprising *N* plants connected by one fungus (which means that each plant is associated with just one fungus). When a plant is attacked, the fungus may monitor cues of herbivore attack produced by the initially attacked plant and signal this information to the other plants it is connected to (recipients). As before, signals may be dishonest and ignored. The difference is that, now, signal investment and signal dishonesty are fungal rather than plant traits (signal response is still a plant trait). We assume that fungi gain a benefit by being connected to plants in better condition (“fitter”), because such plants will be more able to provide the fungus with carbon in exchange for nutrients provided by the fungus (i.e., these plants are “better trade partners”) (35, 36, 77). Specifically, a fungus gains a benefit of *bf_partner_*, where *f_partner_* gives the average condition (fecundity) of the plants it is connected to. Fungi reproduce by producing offspring (juveniles) in proportion to their fecundity; each juvenile migrates to a random deme, and a random juvenile is chosen for each deme to survive and form the next adult fungal population. In SI Appendix, Appendix N, we determined the ESS levels of fungal signaling investment $\left({\tilde{x}}^{*}\right)$ and plant signal response (y*).

#### Fungal monitoring and signaling is evolutionarily stable

We found that mycorrhizal fungi could be selected to monitor plants, to determine whether they are being attacked, and then signal that information to (warn) the other plants they are connected to. We found that fungi could gain a benefit from this, because it helps their trade partners defend themselves against attacks, allowing them to better transfer carbon to the fungus. Mathematically, fungal monitoring and signaling is favored when the costs to the fungus of monitoring and signaling are less than the benefit to the fungus of having higher quality trade partners (Fig. 3B). In contrast, we showed in SI Appendix, Appendix N that fungi are never favored to signal dishonestly, by tricking plants into up-regulating their defenses when no attack is imminent, because this would reduce the quality of their trade partners. Empirically, mycorrhizal fungi are capable of both perceiving cues of herbivore attack and inducing herbivore resistance in the plants they are connected to (16, 78).

One possibility we have not considered is that multiple fungal individuals associate with each other or the same plant (79, 80). Previous theory has shown that, when more fungi and plants interact within a network, this favors more efficient resource trading, and hence helps make trading evolutionarily stable (77). More complex network interactions could similarly influence the evolution of monitoring and signaling by fungi. For example, could larger networks lead to fungi monitoring other fungi, and hence increased selection for fungal signaling? This would be especially the case if it helped facilitate trading across the network. Or could larger networks select for fungal cheating, where some fungi avoid any cost of signaling? Answering such questions would require an alternative modeling approach, that examined more complex networks. Nonetheless, more complex situations do not change that fungi can gain a benefit from monitoring plants and then passing that information to other plants that they trade with.

## Discussion

We have applied a body of signaling theory that has been well developed to explore animal behavior to a different context, information transfer between plants. Our results show that plants: 1) are unlikely to be selected to signal to their neighbors about the presence of herbivores (Fig. 2); 2) can be favored to produce dishonest signals, where they signal an attack when none has occurred, but that this will select for other plants to ignore signals; and 3) can be favored to pay resources to suppress any information (cue) to neighbors about attack (Fig. 3A). In contrast, mycorrhizal fungi can be selected to monitor their host plants, detect when they are attacked, and then signal (warn) other plants in their network (Fig. 3B).

Our results do not support the hypothesis of warning signals by plants, passed via any route, including common mycorrhizal networks or the air (2, 11–24, 27, 81). This is because these warnings would benefit neighboring competitors, to the cost of the signaling individual. Furthermore, we found that not only are plants not expected to signal but that they can be selected to signal dishonestly or to actively suppress any cues of being attacked. Dishonest signaling or suppression of cues is favored to harm or avoid helping neighboring competitors. Empirically, there is little evidence for plant–plant honest signaling (intraspecific), though plants can be favored to signal honestly to their pollinators and seed dispersers (interspecific), who they are not in direct competition with (34, 82–86).

For a helping behavior to be favored, such as signaling a warning of herbivore or pathogen attack, we showed that this would require that helping and competition occur at different scales (economic neighborhoods) or some method of kin recognition / discrimination (30, 31, 46, 47, 55, 87–89). Helping and competition occurring at different scales is relatively unlikely for plants because they are immobile, meaning local interactions involve both cooperation and competition for resources, disfavoring warning signaling (90). This is not always the case in mobile organisms such as animals and bacteria. The same problem of local competition has been demonstrated empirically in other organisms, such as when fig wasps compete for mates in the closed environment of a fig fruit, or when bacteria secrete “public goods” (91–93). However, the problem of local competition can be overcome in mobile organisms, if helping occurs between relatives before they disperse to compete with nonrelatives (94–96). In animals, warnings about the presence of predators have also been argued to be favored because they also reduce predation on the individual making the warning call—this is different from plants, where the warning arises after attack (97–99). In contrast, local competition favors harming behaviors, because harming neighbors can decrease competition for resources (31, 100–102). Alternative modeling approaches to examine these issues could include explicit spatial structures, such as on a graph or lattice, but these have been shown to lead to analogous results (87, 103–105).

Information about herbivore attack could potentially occur through mycorrhizal networks or volatiles in the air (33, 34, 74). The production of herbivore-induced plant volatiles may be unavoidable and does not seem to confer a fitness benefit on the producer (34), leading to the suggestion that herbivore-induced plant volatiles are likely to represent cues rather than signals (62–75). This is consistent with our plant signaling and cue-suppression models, which did not make any assumptions about how information about herbivore attack is transferred. More generally, there may be many ways for information about herbivore attack to be transferred, suppressed, directed toward kin, etc., with some mechanisms more biologically plausible than others (34). Our intention has been to examine the evolutionary stability of different forms of information transfer, in a way that could be applied to a diversity of proximate mechanisms (106).

In contrast to the situation for plants, we found that mycorrhizal fungi can be favored to monitor their host plants, detect a cue of when they are attacked, and then signal this to (warn) other plants in their network (13). Fungi are selected to monitor and signal because defended plants will maintain better condition and hence become better trade partners. Previous theory has shown that selection for fungi and plants to trade resources with each other is increased when multiple plants and fungi interact in the same network, because this stabilizes efficient trading (77).

To conclude, we examined hypotheses explaining the empirical result that, when one plant in a mycorrhizal network is attacked, this leads to other plants in the network up-regulating their defense mechanisms (1–5, 8). Our modeling suggests that this is more likely to represent either a cue produced by plants that is too costly to suppress, or fungi monitoring plants, and then signaling to other plants. Further experiments could test between these possibilities, by examining the underlying mechanism in networks or experimental multiple root systems. How is information conveyed? Where does that information arise from? What are the fitness consequences for all the individuals involved? A greater understanding of these mechanisms could potentially also be exploited in an agricultural context, by facilitating plant defense against herbivores.

## Materials and Methods

In Supplementary Information, comprising SI Appendix, Appendixes A−O, we analyze and interpret a series of models. The models differ from each other in the assumptions they make about: lifecycle; how demography affects relatedness; and what traits can evolve. SI Appendix, Appendixes L and M present our most general plant signaling models, where signals can evolve to be dishonest and / or ignored. SI Appendix,Appendixes A−D present special cases of these models, where signals are forced to be honest and responded-to. SI Appendix, Appendix E presents a model of plant signaling in which plants can recognize their kin. SI Appendix, Appendixes G–J present cue-suppression models. SI Appendix, Appendix N presents fungal monitoring models. SI Appendix, Appendix F provides some illustrative “inclusive fitness” versions of our models. SI Appendix, Appendix K interprets the models presented in SI Appendix, Appendixes A−J, setting them in the context of the wider literature on the evolution of helping and harming. SI Appendix, Appendix O provides some supplementary discussion of how “competition” is modeled.

## Supplementary Material

Supplementary Information

## Figures and Tables

**Fig. 1:**
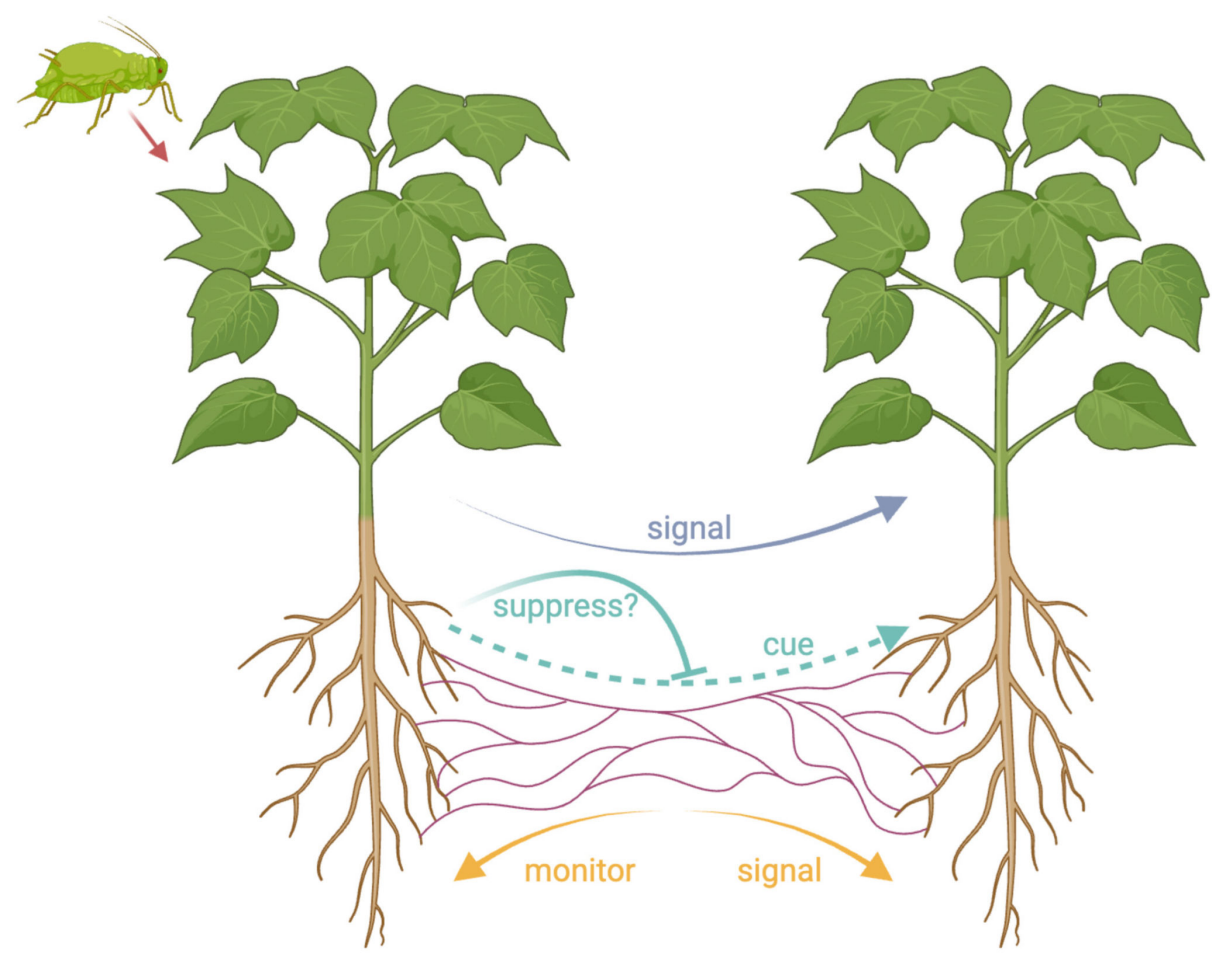
Hypotheses for information transfer in plant–fungal networks. Two plants are connected via a mycorrhizal network. One plant is under attack (e.g., by aphids), and this information may be transferred via the mycorrhizal network to the other plant, allowing it to up-regulate its defense mechanisms. There are three hypotheses regarding how this information is transferred: (blue) signaling by the attacked plant; (cyan) cues, which are potentially vulnerable to suppression by the attacked plant; and (orange) monitoring and signaling by the mycorrhizal network. This figure was made with https://Biorender.com.

## Data Availability

All study data are included in the article and/or SI Appendix.

## Significance

Experiments have shown that when one plant is attacked by a herbivore, this can lead to other plants connected to the same mycorrhizal network up-regulating their defense mechanisms. It has been hypothesized that this represents signaling, with attacked plants producing a signal to warn other plants of impending harm. We found theoretically that plant warning signals are rarely evolutionarily stable. Instead, we identify two viable alternatives that could explain the empirical data: 1) being attacked leads to a cue (information about the attack) which is too costly for the attacked plant to suppress; 2) mycorrhizal fungi monitor the host plants, detect when they are attacked, and then the fungi signal this information to warn other plants in their network.
